# Conical Refraction of Elastic Waves by Anisotropic Metamaterials and Application for Parallel Translation of Elastic Waves

**DOI:** 10.1038/s41598-017-10691-6

**Published:** 2017-08-30

**Authors:** Young Kwan Ahn, Hyung Jin Lee, Yoon Young Kim

**Affiliations:** 1 School of Mechanical and Aerospace Engineering, Seoul National University, 599 Gwanak-ro, Gwanak-gu, Seoul, 151-744 Korea; 2 Institute of Advanced Machinery and Design, Seoul National University, 599 Gwanak-ro, Gwanak-gu, Seoul, 151-744 Korea

## Abstract

Conical refraction, which is quite well-known in electromagnetic waves, has not been explored well in elastic waves due to the lack of proper natural elastic media. Here, we propose and design a unique anisotropic elastic metamaterial slab that realizes conical refraction for horizontally incident longitudinal or transverse waves; the single-mode wave is split into two oblique coupled longitudinal-shear waves. As an interesting application, we carried out an experiment of parallel translation of an incident elastic wave system through the anisotropic metamaterial slab. The parallel translation can be useful for ultrasonic non-destructive testing of a system hidden by obstacles. While the parallel translation resembles light refraction through a parallel plate without angle deviation between entry and exit beams, this wave behavior cannot be achieved without the engineered metamaterial because an elastic wave incident upon a dissimilar medium is always split at different refraction angles into two different modes, longitudinal and shear.

## Introduction

The wave behavior in an anisotropic medium can be quite different from that in an isotropic medium. While a normally incident wave into an isotropic medium travels parallel to the incident direction, it can be multi-directionally deflected in an anisotropic medium such as a biaxial crystal when the so-called acoustic axes^1–3^ (optical axes^4^ for electromagnetic waves) exist. Acoustic and optical axes are the directions in which two or more wave sheets meet in the Equi-Frequency Contour (EFC). This multi-directional refraction phenomenon, known as conical refraction^3–11^, can occur only in anisotropic media. Many interesting applications^12^ have been reported in electromagnetic wave field involving transverse wave modes only, but conical refraction is little explored in elastic waves since they involve dissimilar wave modes, longitudinal and shear wave modes, not to mention actual realization and experiment. We present the realization of the phenomenon and achieve parallel wave translation with elastic waves for the first time as supported by the experiments.

Because it is difficult to utilize conical refraction phenomenon only using natural materials even for electromagnetic wave manipulation, considerable efforts have been made by using metamaterials. Metamaterials have been applied for various types of wave manipulation such as negative refraction^13^, wave focusing^14^, and hyperlens^15^. Now, for instance, conical refraction can be achieved by using biaxial hyperbolic metamaterials that have a negative value in one of dielectric constants along three principal directions^16^. Furthermore, a metamaterial composed of a pseudochiral medium with a chirality parameter allows normally incident waves to deflect^17, 18^. In addition, conically refracted waves have been demonstrated via numerical simulation by designing a specific EFC shape based on a bi-anisotropic metamaterial^19^. These studies showed that electromagnetic waves can be deflected as they propagate into anisotropic metamaterials having specific effective properties. Compared to the progress in electromagnetic metamaterials for conical refraction, there is little progress in elastic metamaterials demonstrating conical refraction even with its practical usefulness in the fields of nondestructive testing^20^, structural health monitoring^21^, and ultrasonic imaging techniques^22, 23^. The intrinsic difficulty in elastic waves is due to the co-existence of dissimilar wave modes, longitudinally-polarized and transversely-polarized and their coupling. Therefore, unless the mode coupling and dissimilar polarization are fully investigated, it is difficult to engineer such anisotropic elastic metamaterials. In this respect, our unique contribution is to design elastic anisotropic metamaterials that exhibit conical refraction.

**Figure 1:**
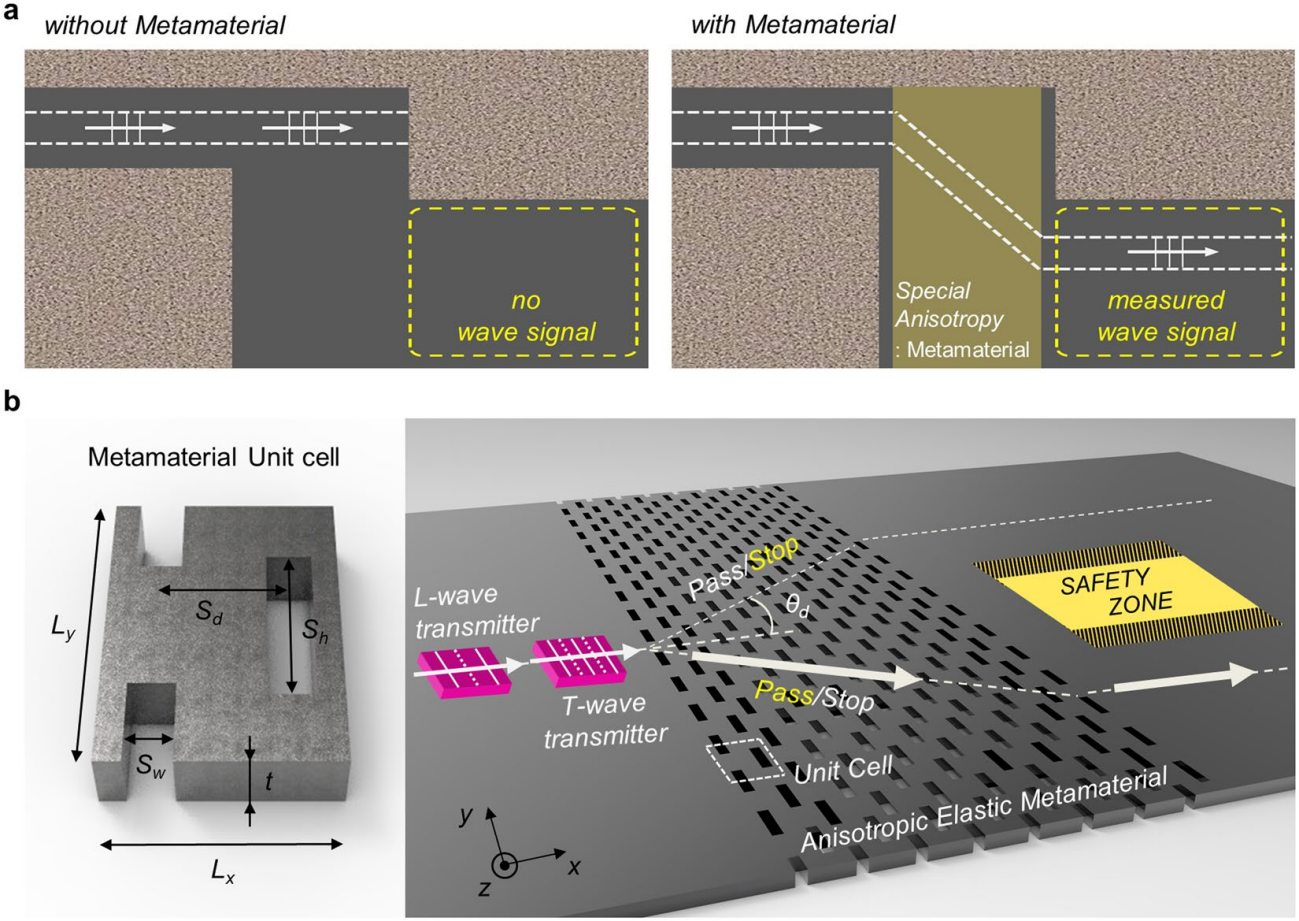
The engineered metamaterial for parallel translation of elastic waves. (**a**) Wave propagation paths with and without the target anisotropic system. (**b**) Unit cell dimensions of the structured anisotropic metamaterial plate with thickness *t* (left) and the proposed methodology for the parallel translation of mechanical waves based on two types of transmitters (right) (*L*_*x*_ and *L*_*y*_ lattice period along *x* and *y* directions, S_*w*_ and S_*h*_ width and height of slit, and S_*d*_ the distance between adjacent slits along *x* direction).

As a specific motivation to realize conical refraction involving elastic waves, one could envision ultrasonic non-destructive testing of a system with obstacles, as depicted in Fig. 1a. In Fig. 1, the region inside the yellow dotted box is to be inspected by the ultrasound inputted along the path marked by arrows and the patterned brown region represents obstacles. A horizontally incident elastic wave cannot reach the inspection region, as shown in the left illustration of Fig. 1a. On the other hand, it can reach the inspection region if the incident wave is deflected into an anisotropic slab made of an elastic metamaterial, as shown in the right illustration of Fig. 1a. We will show that the deflection can be possible only if the elastic metamaterial can result in conical refraction inside it. It will be also shown that two phase-tuned elastic waves of different modes, longitudinal and transverse (i.e., shear) modes, must be simultaneously transmitted into the metamaterial to achieve the wave translation depicted in the right illustration of Fig. 1a. The wave path inside the metamaterial demonstrating conical refraction is depicted by a big arrow in Fig. 1b. This wave transmission aspect depicted in Fig. 1b may be referred to as the parallel translation of an elastic wave through the anisotropic metamaterial slab. Apparently, this parallel translation resembles an obliquely-incident light passing through a parallel glass plate without angle deviation between entry and exit beams in air. Since an elastic wave incident upon a dissimilar medium in a plane is always split into two different modes, longitudinal and transverse, at different refraction angles, the parallel translation of waves in the elastic field is different from that in the electromagnetic field. Therefore, there has been no successful report on the parallel translation of elastic waves not to mention the actual realization of conical refraction with elastic waves.

The first part of this paper will be focused on the design and realization of an anisotropic elastic metamaterial that realizes conical refraction at a target frequency range. The underlying condition for conical refraction in the elastic regime is that the longitudinal modulus must be equal to the shear modulus^5^. Considering the fact that shear modulus is always smaller than longitudinal modulus in isotropic media, this calls for special anisotropic media, which may not be possible without artificial metamaterials. In our study, we propose a single-phase metamaterial made from aluminum, an isotropic medium. In particular, we fabricated a metamaterial slab in a thin aluminum plate such that its effective longitudinal and shear moduli are the same. This condition is equivalent to the condition for equal longitudinal and shear wave speeds. Accordingly, the unit cell of the metamaterial is so designed that it retards its longitudinal speed more than its shear wave speed relative to the wave speeds in the base aluminum plate.

As waves in a plate are considered, the symmetric Lamb wave and guided shear-horizontal wave are used to simulate the longitudinal and transverse waves, respectively, in bulk media. Using the designed metamaterial slab demonstrating conical refraction, we first show that a horizontally incident longitudinal wave is split into two coupled longitudinal-transverse waves inside the metamaterial slab. Then, the two waves exit the metamaterial slab as two horizontal beams, which are sketched with two dotted lines in Fig. 1b. Interestingly, splitting into two parallel waves can cloak^24, 25^ an object against a horizontally incident wave if the designed metamaterial slab is placed in front of it. We will also show that the designed metamaterial in the left illustration of Fig. 1b has a very wide operating frequency range because it is a non-resonant type. The bi-deflection wave phenomenon will be investigated using simulations and experiments.

After performing the bi-deflection of a single incident wave, we further investigate the phase information of the two deflected waves critical in realizing the parallel translation of elastic waves in Fig. 1a. The investigation of the phase information from the simulation and experiment of conical refraction leads to an important finding where upon an incidence of a longitudinal wave into the metamaterial, the two exiting longitudinal waves are in phase while the two exiting transverse waves are out of phase. On the other hand, upon an incidence of a transverse wave into the metamaterial, the two exiting transverse waves are in phase while the two exiting longitudinal waves are out of phase. By using this phase information, one could possibly develop a wave excitation method using two transducers, one generating a longitudinal wave and the other generating a shear wave. If they are properly phase-controlled based on the finding of the above-mentioned phase information, the parallel translation can be either upward or downward. We will give the details of this technique in the paper. The realization of the parallel translation of elastic waves will be demonstrated both numerically and experimentally and its potential applications will be also discussed.

## Results

### Metamaterial realization for conical refraction of elastic waves

**Figure 2:**
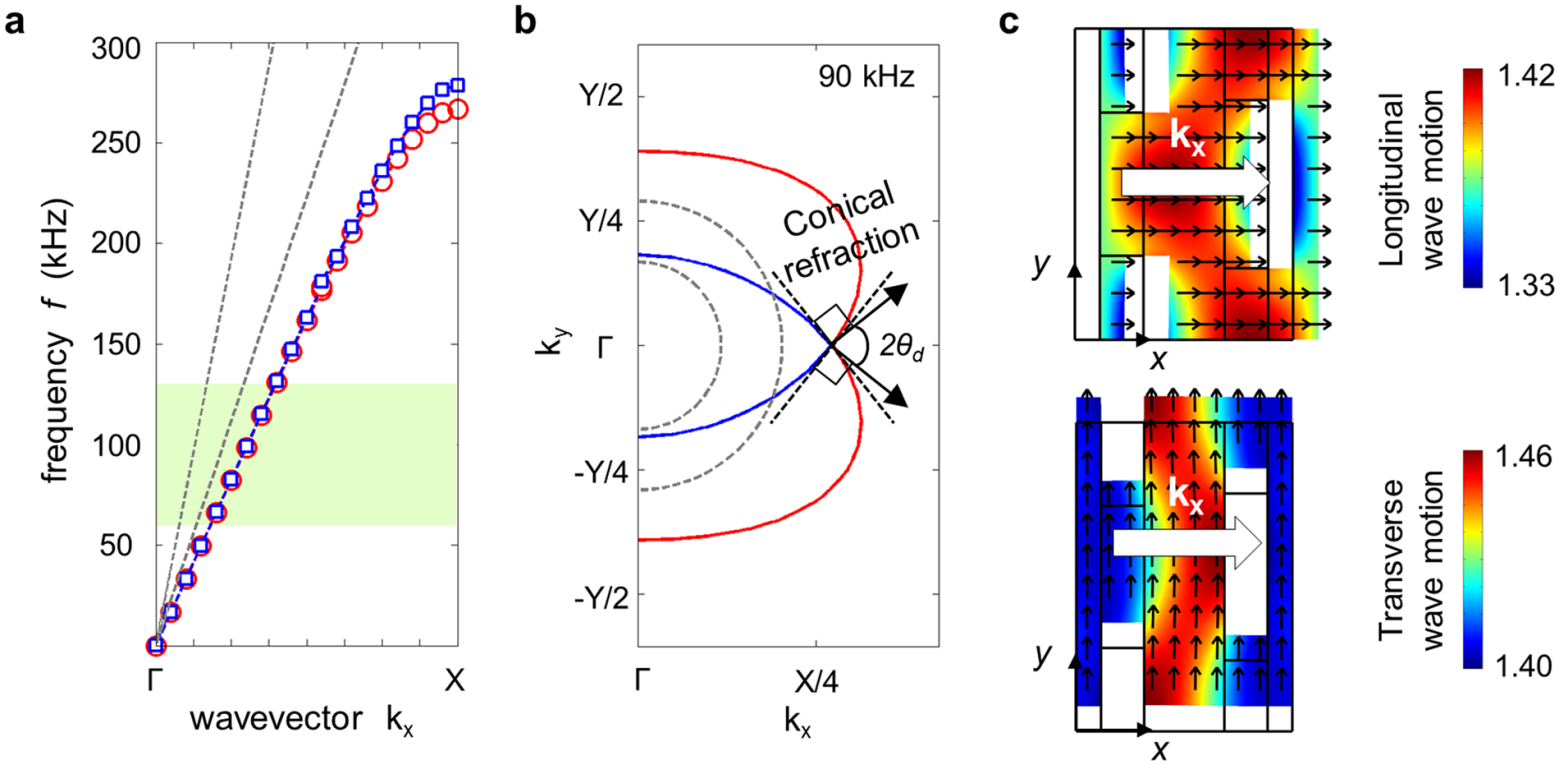
Wave characteristics in the designed metamaterial for conical refraction. (**a**) The dispersion curves and (**b**) equi-frequency contours at 90 kHz of a homogeneous 1-mm thick aluminum plate and the 1-mm thick engineered metamaterial plate designed in Fig. 1b. (**c**) Mode shapes of the longitudinal and transverse wave motion for waves propagating along the *x* axis. In (**a** and **b**) blue and red solid lines denote the longitudinal and transverse waves in the metamaterial plate, respectively. The two grey dashed lines denote longitudinal and shear waves in an aluminum plate (the longitudinal wave has the steeper slope in (**a**) and is represented by the smaller semi-circle in (**b**)).

As reported earlier^5^, conical refraction for elastic waves occurs if *C*_11_ = *C*_66_ and *C*_16_ = *C*_26_ = 0 where *C*_*ij*_ denotes the component of the stiffness tensor **C**. Referring to Figs 1b and 2, *C*_11_ represents the longitudinal bulk modulus along the *x* axis while *C*_66_ is the shear modulus in the *x* − *y* coordinate system. Because the phase speeds of longitudinal and transverse waves are given by ${V}_{L}=\sqrt{{C}_{11}/\rho }$ and ${V}_{T}=\sqrt{{C}_{66}/\rho }$ (*ρ*: density), respectively, the relation of *C*_11_ = *C*_66_ implies that *V*_*L*_ should be equal to *V*_*T*_. Focusing on waves propagating along the Γ − X direction (equivalently, along the *x* direction) for a range of frequencies between 60 kHz and 120 kHz (with the specific target frequency of 90 kHz), the metamaterial for conical refraction is designed with the proposed unit cell shown in Fig. 1b. The metamaterial is single-phased because it is fabricated by machining an aluminum plate of 1 mm in thickness. Because *V*_*L*_ is always faster than *V*_*T*_ in a homogenous isotropic medium, the metamaterial must have some geometrical features that can slow down the longitudinal wave speed along the Γ − X direction. To fulfill this requirement, off-centered vertical rectangular slits are inserted in the unit cell of the designed metamaterial, as motivated by Lee^26^. The selected base material for the metamaterial design is aluminum (density *ρ* = 2700 kg/m^3^, Young’s modulus *E* = 69 GPa, Poisson’s ratio *ν* = 0.33) with *V*_*L*_ = 5355.2 m/s and *V*_*T*_ = 3099.6 m/s. Clearly, these slits slow down the longitudinal wave speed in the *x* direction while they would not affect the shear wave speed much. The wave behavior in the designed metamaterial will be investigated below.

First, we will check if the phase velocities *V*_*L*_ and *V*_*T*_ of the longitudinal and transverse waves in the designed metamaterial are indeed the same at the target frequency. To this end, we calculate the effective density *ρ* and stiffness components *C*_*ij*_ by using the S-parameter retrieval method^27^. The results are listed in Table 1 for the frequency range of interest. In the present study, the unit cell of the elastic metamaterial mainly targeted at 90 kHz is configured as: (referring to Fig. 1b)

$$\begin{array}{c}\begin{array}{l}{\rm{Lattice}}\,\text{periods}:\,{L}_{x}=3.5\,{\rm{mm}}\,{\rm{and}}\,{L}_{y}=5\,{\rm{mm}}\\ {\rm{Slit}}\,\text{dimension}:\,{S}_{w}=0.7\,{\rm{mm}}\,{\rm{and}}\,{S}_{h}=2.7\,{\rm{mm}}\end{array}\\ {\rm{Gap}}\,{\rm{between}}\,{\rm{adjacent}}\,\text{slits}:\,{S}_{d}=2.0\,{\rm{mm}}.\end{array}$$

**Table 1:** Effective density and stiffness of the designed elastic metamaterial for conical refraction in a certain frequency range.

Frequency (kHz)	ρ (kg/m^3^)	C_11_ (Pa)	C_12_ (Pa)	C_22_ (Pa)	C_66_ (Pa)	C_16_, C_26_ (Pa)
60	2070.0	1.099 × 10^10^	6.230 × 10^9^	5.066 × 10^10^	1.098 × 10^10^	0
70	2057.0	1.090 × 10^10^	6.171 × 10^9^	5.034 × 10^10^	1.089 × 10^10^	0
80	2045.6	1.081 × 10^10^	6.084 × 10^9^	5.015 × 10^10^	1.080 × 10^10^	0
90	2035.7	1.072 × 10^10^	5.994 × 10^9^	5.004 × 10^10^	1.072 × 10^10^	0
100	2026.3	1.064 × 10^10^	5.921 × 10^9^	4.989 × 10^10^	1.064 × 10^10^	0
110	2018.7	1.056 × 10^10^	5.846 × 10^9^	4.981 × 10^10^	1.055 × 10^10^	0
120	2014.8	1.050 × 10^10^	5.787 × 10^9^	4.985 × 10^10^	1.048 × 10^10^	0

In the frequency range of interest centered at 90 kHz, the long wavelength assumption for the metamaterial is valid because the size of the metamaterial unit cell is approximately 0.13*λ* at 90 kHz. Therefore, the effective medium theory can be used. Using the formulas ${V}_{L}=\sqrt{{C}_{11}/\rho }$ and ${V}_{T}=\sqrt{{C}_{66}/\rho }$ based on the effective material properties in Table 1, the wave speeds at *f* = 90 kHz are found to be metamaterial plate:

$${V}_{L}=2294.8\,m/s={V}_{T}=2294.8\,m/s\,{\rm{at}}\,f=90\,{\rm{kHz}}.$$

These numerical values confirm that the designed metamaterial plate has the same longitudinal and transverse phase velocities at the target frequency, satisfying the condition for the conical refraction of elastic waves.

To further explore the wave characteristics of the engineered metamaterial, its dispersion curve and equi-frequency contour are plotted in Fig. 2. The finite element analysis was used (the same contours can be obtained by solving the Christoffel equation^28^ as given in Supplementary Materials). The lowest symmetric Lamb wave mode (S_0_) simulating the bulk longitudinal wave and the shear-horizontal wave mode (SH_0_) simulating the bulk shear wave are considered for the plot. There also exists the lowest antisymmetric Lamb wave mode (A_0_) but this mode is not of our concern because it cannot be used for conical refraction. Figure 2a shows that the values of *f*/*k*_*x*_ (*k*_*x*_: wave number in the *x* direction) for the two branches (red and blue lines) of the metamaterial are virtually the same for a wide frequency range from 60 kHz to 120 kHz, implying *V*_*L*_ ≈ *V*_*T*_. The wide range of working frequencies is due to the fact that the designed metamaterial is a non-resonant type metamaterial.

Now let us focus on conical refraction by examining the EFC plotted in Fig. 2b. Two EFC’s of the metamaterial that correspond to two sheets of longitudinal and transverse wave modes, as denoted by blue and red lines in Fig. 2b, intersect at *k*_*y*_ = 0. If a wave is incident from an isotropic medium into the metamaterial along the *x* direction (*k*_*y*_ = 0 and *k*_*x*_ ≠ 0), the *k*_*y*_ component must be preserved. Thus, two wave modes of the metamaterial that co-exist at the intersection point can be excited simultaneously. Figure 2b also shows that the horizontally incident wave (along the *x* direction) is deflected into two waves with the deflection angles of ±*θ*_*d*_. For the selected geometry of the unit cell, *θ*_*d*_ is found to be 37.9°. Note that *θ*_*d*_ is governed^7^ by the anisotropy factor *A* = 2*C*_66_/(*C*_11_ − *C*_12_). The effects of the material properties are further investigated in Supplementary Materials. The mode shapes representing particle motions at the intersection point are shown in Fig. 2c. In the figure, big open arrows denote the propagation direction of the phase velocity and the black solid lines indicate the particle motion directions. The line lengths correspond to the particle displacement magnitudes. The magnitudes are also presented in color level on the deformed shapes for clarity.

We will now examine how *V*_*L*_ can become equal to *V*_*T*_ in the designed metamaterial. Note that *C*_16_ = *C*_26_ = 0 can be automatically satisfied if the metamaterial unit cell has horizontal symmetry. Keeping in mind that the longitudinal wave speed is faster than the shear wave speed in the base isotropic medium (without any slit), we will carefully examine the mode shapes at the intersection of the two EFC’s of the metamaterial. The top and bottom illustrations in Fig. 2c show the mode shapes of the dominant longitudinal and transverse wave modes. Considering wave motion in the central part of the unit cell for the longitudinal mode, a wave cannot pass directly along the horizontal line from the left end to the right end due to the large vertical void slit (the waves incident from the upper and lower parts of the unit cell cannot pass freely along the *x* axis, because the two vertical slits are off-center from the large vertical slit near the right end of the unit cell). Accordingly, the waves must detour upwards and downwards to reach the right end. As a result, the actual path of the traveling wave is elongated, which slows the effective propagating wave speed in the unit cell. This phenomenon may be compared to the coiling-up space phenomenon reported earlier in acoustic problems^29^. On the other hand, the situation for the transverse wave mode is quite different as shown in the bottom illustration in Fig. 2c. The transverse wave causing particles to move vertically can propagate from the left end to the right end of the unit cell without significantly hindering the vertical motions. Consequently, the transverse wave speed in the metamaterial becomes less affected than the longitudinal wave speed. Using this mechanism, the size and location of the off-centered slits are selected to make *V*_*L*_ = *V*_*T*_.

**Figure 3:**
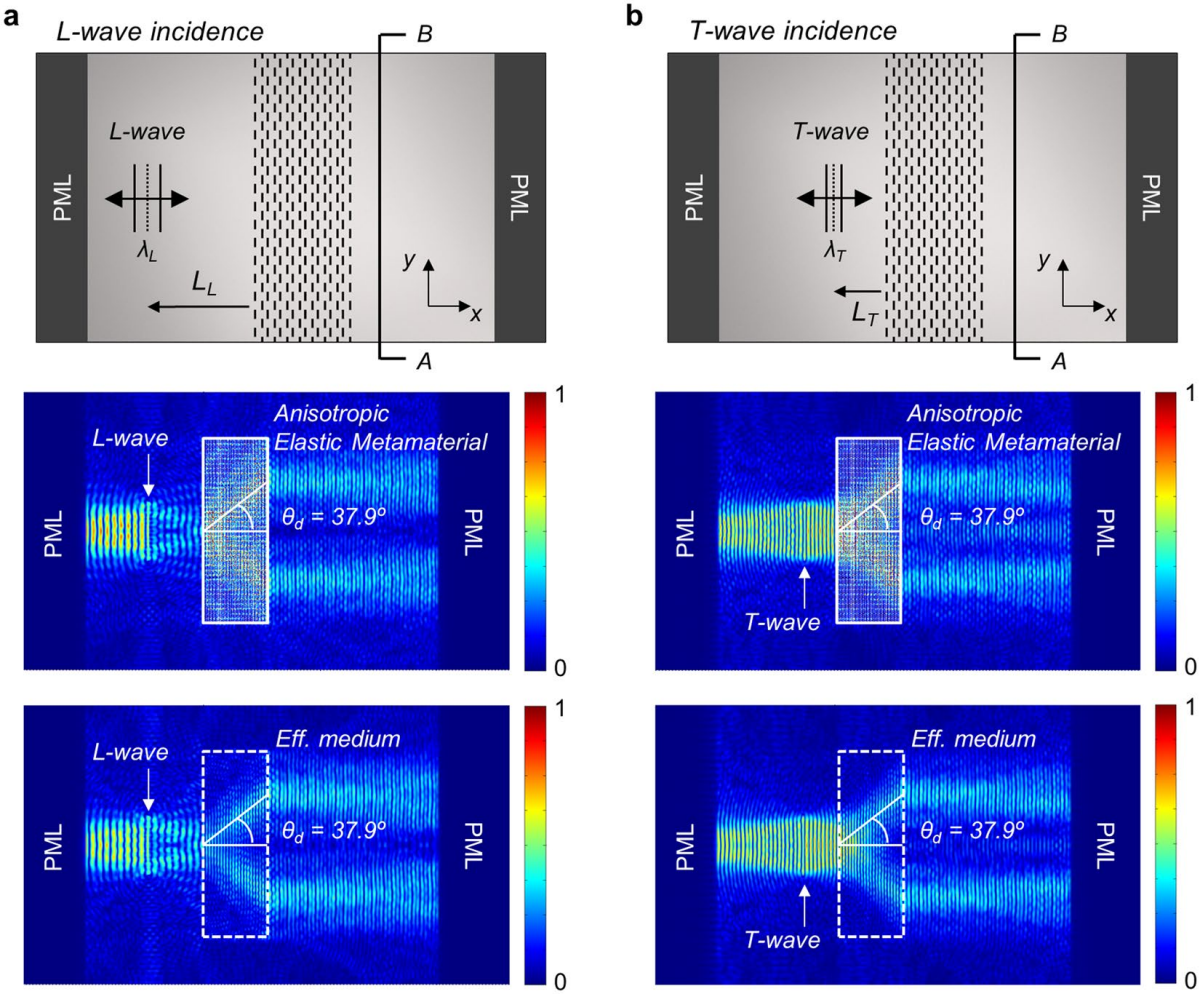
Numerical simulation of the conical refraction phenomenon occurring in the designed anisotropic metamaterial. (**a**) Longitudinal and (**b**) transverse wave incidences at 90 kHz. Normalized stress fields are plotted in the middle and bottom plots. The regions surrounded by the solid and dashed white boxes are filled with the anisotropic metamaterials with the detailed unit cell configurations and the effective anisotropic medium, respectively.

The finite element simulation results for the conical refraction with the designed metamateral are shown in Fig. 3. COMSOL Multiphysics is used for simulations conducted with the plane stress assumption in this study. As depicted in Fig. 3a and b, the plane wave sources for longitudinal waves (L-wave) of wavelength *λ*_*L*_ = 59.5 mm or transverse waves (T-wave) of wavelength *λ*_*T*_ = 34.4 mm at *f* = 90 kHz were assumed to be normally incident from the base aluminum plate to the metamaterial slab along the *x* direction. To absorb boundary reflection from both the right-end and left-end boundaries, Perfectly Matched Layers (PMLs) were applied. The vertical length of the wave was chosen to be 250 mm, which is equivalent to approximately 4*λ*_L_. Below the sketch of the simulation model in Fig. 3a and b, two simulation results are presented in Fig. 3a and b. The metamaterial slab regions of 280 mm × 800 mm are surrounded by the solid white boxes. The color level corresponds to the magnitude of the normalized stress fields. The simulation results with “Anisotropic Elastic Metamaterial” are obtained by using the detailed unit cell model where the metamaterial slab region is filled with 80 × 160 unit cells. On the other hand, the simulation results with “Effective Medium” are obtained by using a homogeneous medium model with the effective material properties given in Table 1. Because the results with “Effective Medium” virtually match those with “Anisotropic Elastic Metamaterial,” we will not distinguish the two results in subsequent discussions.

The simulation results in Fig. 3 obtained by time-harmonic analysis clearly show the conical refraction; a wave incident on the metamaterial slab, either longitudinal or transverse, is deflected at the deflection angle of *θ*_*d*_ = 37.9° into two waves. They exit the metamaterial slab and enter the base aluminum plate parallel to the incidence direction (*x* direction). The angle *θ*_*d*_ = 37.9° shown in the figures was directly calculated from the finite element wave simulations and it is exactly matched with the value calculated from the EFC in Fig. 2b. The time-transient finite element analysis (see Supplementary Materials) predicts the same results as the time-harmonic analysis. However, the transient analysis shows the actual propagation aspect of an incident wave into the metamaterial. In addition, we can quantify the power transmission through the metamaterial slab as detailed in Supplementary Materials. Since a single horizontally incident wave is split into two horizontally propagating waves kept apart by some vertical distance, the conical refraction phenomenon can be used to cloak an object. For instance, the object can be hidden by putting it between two metamaterial slabs and a cloaking example is given in Supplementary Materials.

**Figure 4:**
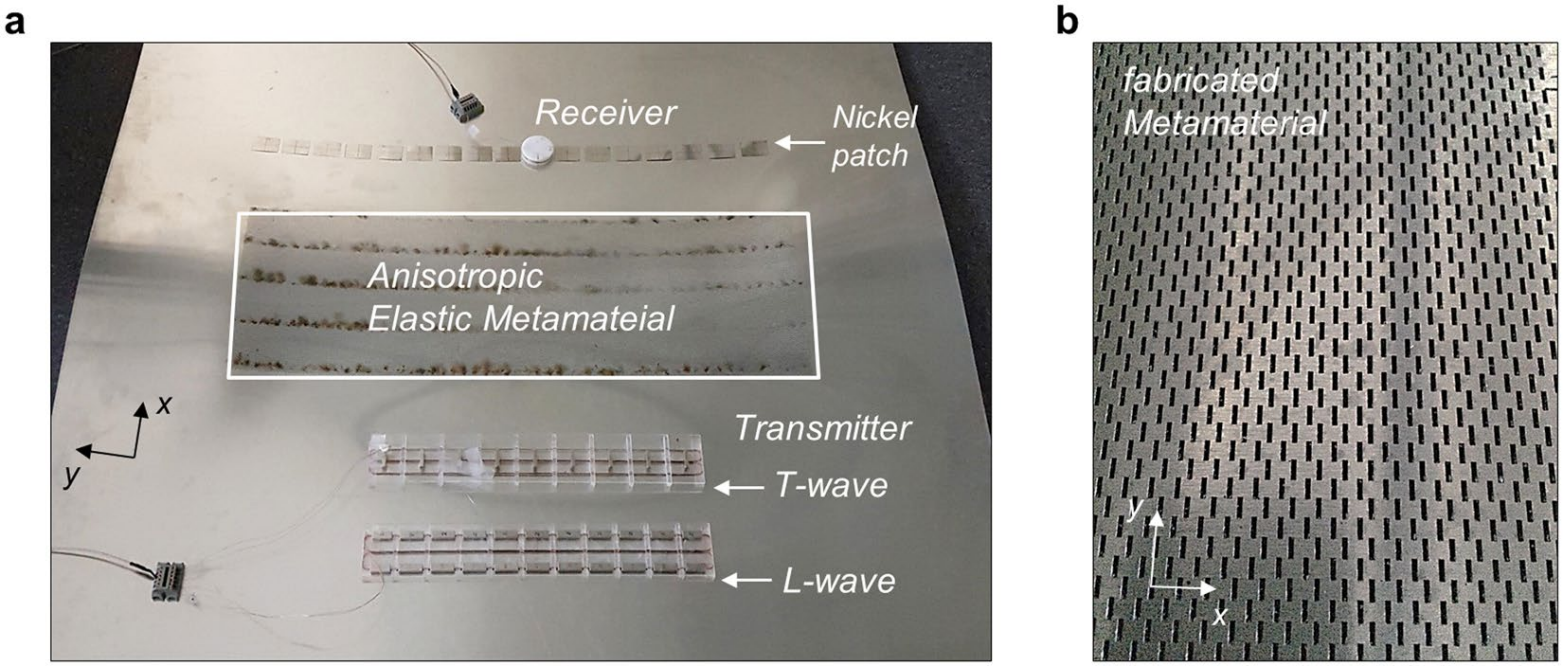
Experimental setup with the proposed metamaterial slab (280 × 800 × 1 mm^3^) for conical refraction (the base aluminum plate is 2400 × 1200 × 1 mm^3^). (**a**) Photo of the experimental setup showing the metamaterial slab fabricated onto an aluminum plate and transmitting and receiving magnetostrictive patch transducers using nickel patches. (**b**) Zoomed-in view of the fabricated metamaterial.

For the experimental validation of the occurrence of conical refraction, the elastic metamaterial was microfabricated onto an aluminum plate by using a laser-cutting machine (TRUMPF TruLaser 5030 fiber). The experimental setup is shown in Fig. 4a. The metamaterial slab in Fig. 4b was patterned with 80 × 160 unit cells in the *x*-*y* plane. As a transmitter, a magnetostrictive patch transducer^30–32^ was used to excite a longitudinal or transverse wave. Its width (along the *y* direction) was sufficiently large (250 mm) enough for a plane wave to be generated. The transducer operates by the magnetostrictive phenomenon, which is the coupling effect between magnetic field and mechanical field (see, e.g., ref. 30). The transducer consists of a thin magnetostrictive patch, coil, and magnets. A similar transducer was successfully used earlier^32^ to generate plane waves, so its detailed operation mechanism will not be repeated here. The distance from one side of the metamaterial to the L-wave (T-wave) transducer is *L*_*L*_ = 4*λ*_*L*_ = 238 mm (*L*_*T*_ = 4*λ*_*T*_ = 138 mm). These *L*_*L*_ and *L*_*T*_ values were chosen to be identical to those used in numerical simulations. The wave that passed through the metamaterial slab was received by a different type of magnetostrictive patch transducer, similar to the OPMT^30^. Depending on the magnetic circuit placed over a magnetostrictive patch, the L-wave or T-wave can be measured selectively^30^. Along the line on the base aluminum plate that is 100 mm away from the wave exiting side of the metamaterial, 17 measurements were made at every 50 mm.

**Figure 5:**
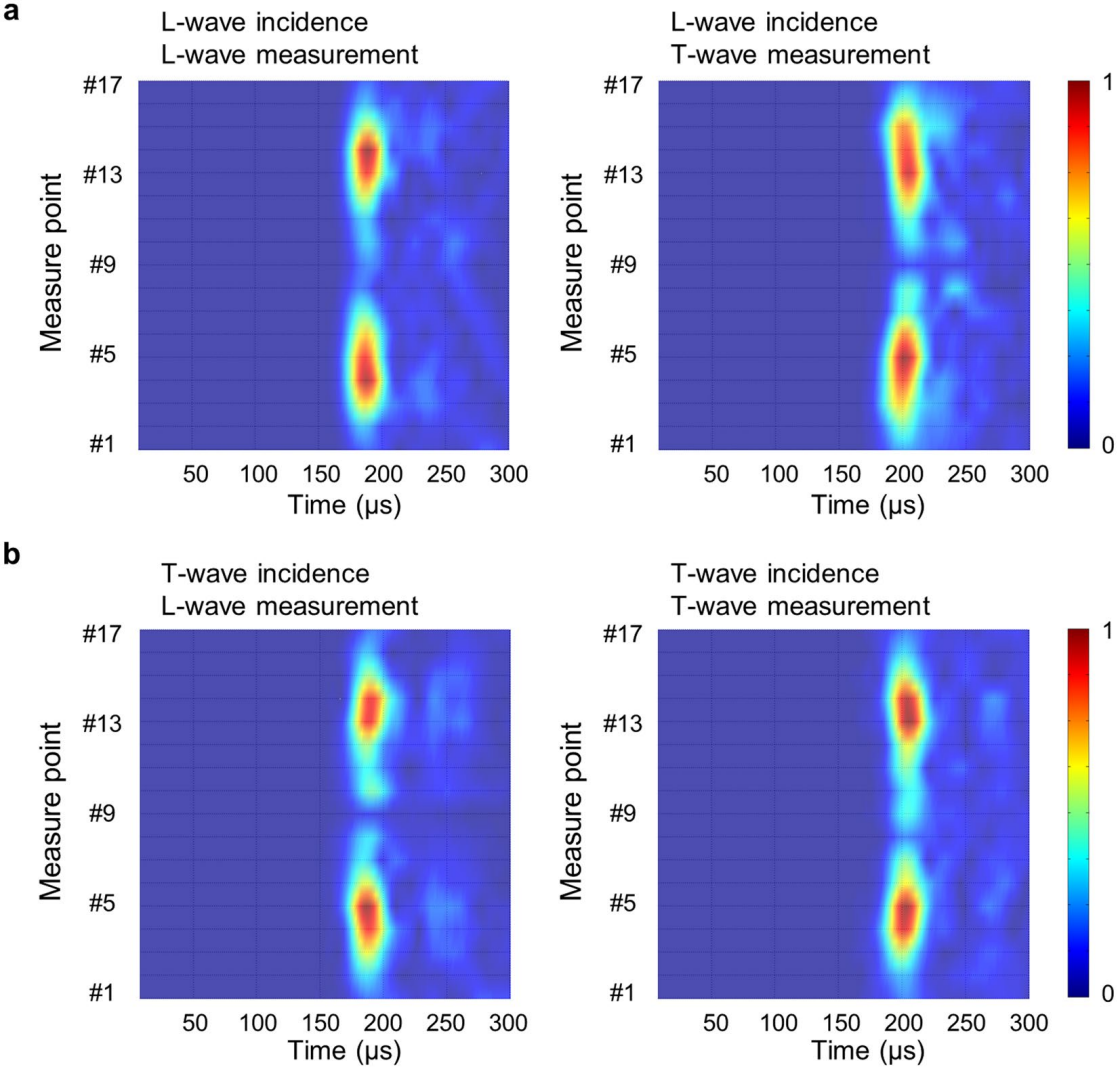
Short-time Fourier Transforms (STFT’s) of the measured signals by the receivers. (**a**) Longitudinal wave incidence of a 90 kHz Gabor pulse. Left: STFT of the measured longitudinal wave, Right: STFT of the measured transverse wave. (**b**) Transverse wave incidence of a 90 kHz Gabor pulse. Left: STFT of the measured longitudinal wave, Right: STFT of the measured transverse wave.

Figure 5 shows the Short-Time Fourier Transform (STFT) of the measured signals by the receiver. The major findings can be summarized by

1. A single horizontally incident wave on the metamaterial that initially propagates toward the central region (aligned with #9), regardless of its wave mode, is split into two waves passing through #5 and #13 (showing the maximum intensity in the plots). This confirms the occurrence of conical refraction through the designed metamaterial.
2. Even if a single-mode wave (longitudinal or transverse) is incident upon the metamaterial, two dissimilar wave modes, longitudinal and transverse, always exit from the metamaterial.

To explain Finding 2, we note that the incident single wave is split into two coupled longitudinal and transverse waves. While the longitudinal and transverse waves have the same speed inside the metamaterial (because of *C*_11_ = *C*_66_), they travel at different speeds when they exit to the base aluminum plate. Therefore, the longitudinal and shear waves under the L-wave incidence were recorded at *t* = 190 *μs* and *t* = 210 *μs*, respectively. The arrival times were found to agree well with those predicted by the transient analysis (see Supplementary Materials). We also remark that the conical refraction with the designed metamaterial occurs over a wide frequency range. In fact, it occurs for the frequency range from 60 kHz to 120 kHz, as demonstrated in Supplementary Materials (see also Fig. 2a). This broadband performance is achieved because the material property condition (C_11_ = C_66_) for conical refraction, causing longitudinal and transverse modes overlapped on the dispersion curve (see Fig. 2), remains to be valid almost up to 150 kHz. It was also confirmed by experiments.

In the next section, we will investigate how the conical refraction can be utilized to achieve the parallel translation of elastic waves, as sketched in Fig. 1a. Because an incident wave is always split into two waves, a technique that cancels the wave deflecting upwards (downwards) and maintains the wave deflecting downwards (upwards) should be devised. Furthermore, the each of the upward and downward waves has two wave modes when they exit to the base plate. This should be carefully considered for the parallel translation.

### Parallel translation of elastic waves

In this section, the parallel translation of elastic waves will be presented. This work can lead to elaborate wave manipulation for non-destructive testing in harsh environment.

**Figure 6:**
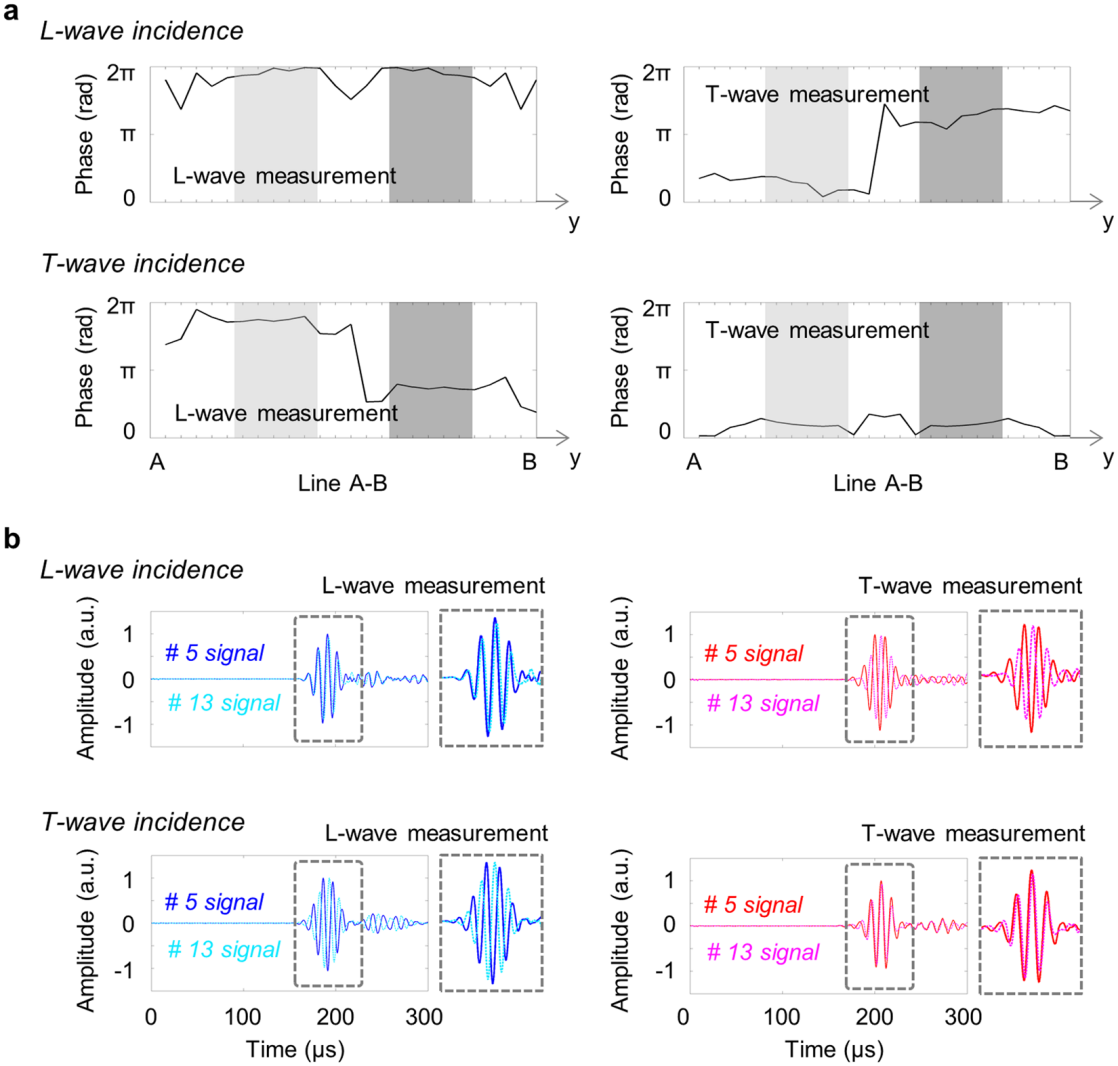
Phase analysis of the signals obtained for verifying the conical refraction phenomenon. (**a**) Phase variation of the calculated signal using the finite element method along the line A-B as indicated in Fig. 3a and b. For the L- and T-wave incidences, the phase distributions for the measured (calculated) L- and T-waves are plotted. The phase information in the two grey regions is the main concern. (**b**) Comparison of the experimentally-measured signals along the receiving line shown in Fig. 4a. Depending on the incident wave mode, the phases measured at #5 and #13 are different even if the same wave mode is measured.

As mentioned previously, the main difficulty with elastic waves in realizing the parallel translation is due to the existence of dissimilar wave modes propagating at different wave speeds. Therefore, the parallel translation of elastic waves cannot be realized with only a single wave input, i.e., only with a single transducer. Thus, we propose a mode-adjusted, phase-tuned wave excitation method using two transmitters. To implement this technique, we must understand the characteristics of the waves that exit to the based material. Figure 6a shows the simulation results for the phase information of the measured longitudinal and transverse waves along the line A-B illustrated in Fig. 3a and b. The results for both the longitudinal and transverse wave incidences are presented. On the other hand, Fig. 6b provides the phase information of the experimentally measured L- and T-wave signals at some measurement locations (#5 and #13) along the receiving line as shown in Fig. 4a. From the simulation and experiment results, the following important observations can be summarized:

1. For the L-wave incidence to the metamaterial, the exited L-wave from the upwardly deflected (or simply upward) wave is *in phase* with that from the downwardly deflected (or simply downward) wave. The exited T-wave from the upward wave is *out of phase* with that from the downward wave.
2. For the T-wave incidence to the metamaterial, the exited L-wave from the upward wave is *out of phase* with that from the downward wave. The exited T-wave from the upward wave is *in phase* with that from the downward wave.

These two observations are critical in realizing the parallel translation. If the L-wave and T-wave are incident simultaneously on the metamaterial, the following consequences can be predicted:

1. If the downwardly exiting L-wave from the L-wave incidence interferes constructively with the downwardly exiting L-wave from the T-wave incidence, the upwardly exiting L-wave from the L-wave incidence interferes destructively with the upwardly exiting L-wave from the T-wave incidence.
2. The same phenomenon occurs for the exiting T-waves.

These consequences imply that if the downwardly exiting longitudinal (or shear) wave from the L-wave incidence is tuned to interfere constructively with the downwardly exiting longitudinal (or shear) wave from the T-wave incidence, the incident waves can always be deflected downward alone and exit the metamaterial parallel to the incident direction. On the other hands, if the downwardly exiting longitudinal (or shear) wave from the L-wave incidence is tuned to interfere destructively with the downwardly exiting longitudinal (or shear) wave from the T-wave incidence, the incident waves can always be deflected upward alone and exit the metamaterial parallel to the incident direction. Therefore, one can realize the upward or downward parallel translation of elastic waves by exiting both L-wave and T-wave simultaneously with proper phase adjustment with using constructive or destructive interference inside the metamaterial.

**Figure 7:**
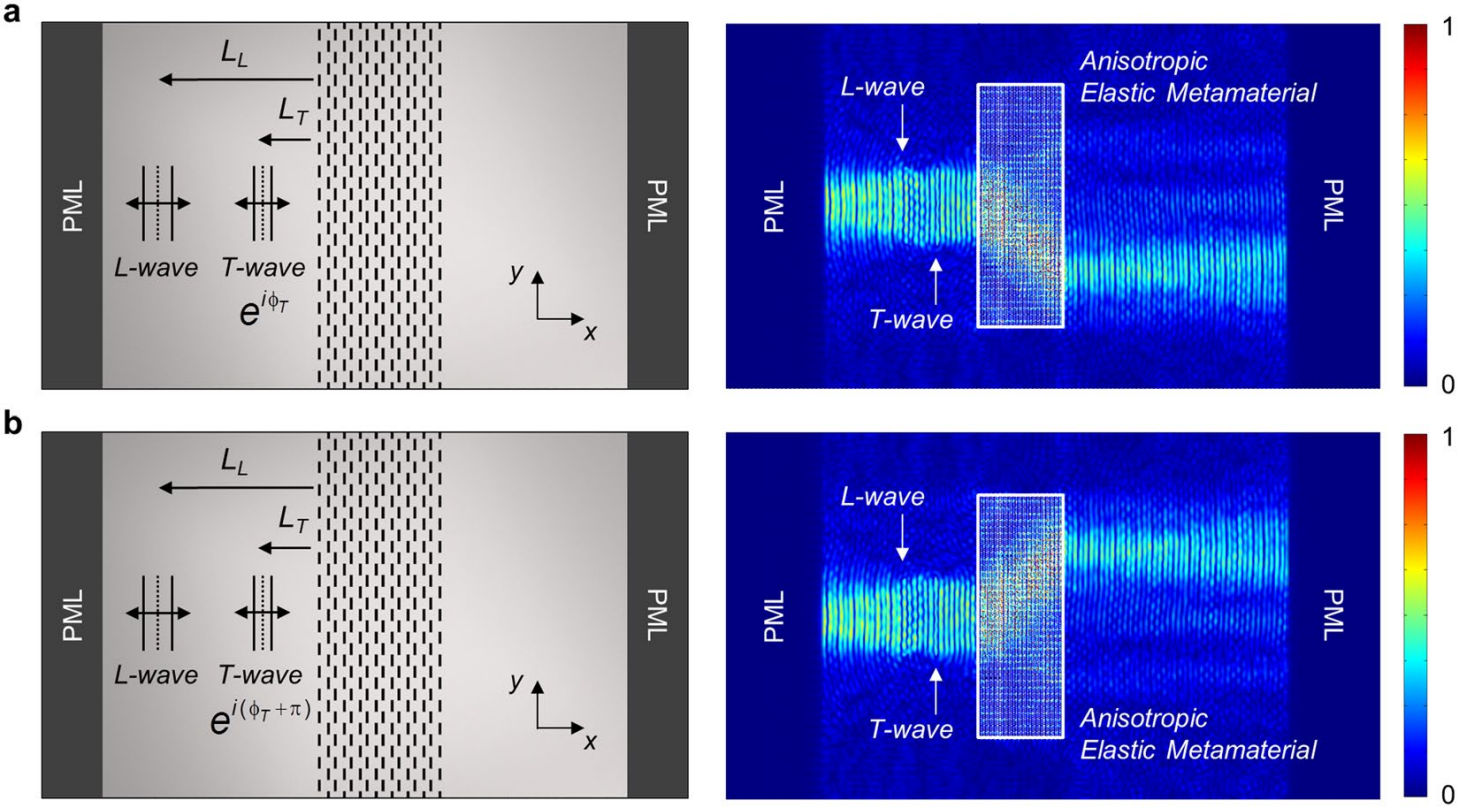
Numerical simulations for the parallel translation of 90 kHz elastic waves using two phase-tuned transducers. (**a**) Parallel downward translation. Left: sketch of simulation model indicating the phase delay of *ϕ*_*T*_ between the two transducers, Right: normalized stress distributions. (**b**) Parallel upward translation. Left: sketch of simulation model indicating the phase delay of *ϕ*_*T*_ + *π* between the two transducers, Right: normalized stress distributions.

Figure 7 shows the two transducers installed for numerical simulations of the parallel translation. The transducer generating the T-wave is placed closer to the metamaterial than the transducer generating the L-wave because the wave speed of the T-wave is slow. If the distances *L*_*L*_ and *L*_*T*_ from the transducers closer to the metamaterial *L*_*L*_ = 4*λ*_*L*_ = 238 mm and *L*_*T*_ = 4*λ*_*T*_ = 138 mm, the excited 90 kHz centered longitudinal and transverse waves reach the metamaterial simultaneously (*λ*_*L*_ and *λ*_*T*_: the wavelengths of the L- and T-waves at 90 kHz). The phase delay of the excited T-wave relative to the excited L-wave is denoted by *ϕ*_*T*_ when constructive interface occurs in the downwardly deflected wave. In this case, the combined wave from the L-wave and T-wave transmitters is translated downwardly and exits the metamaterial as if it were parallel translated; see Fig. 7a. If the phase delay of the excited T-wave related to the excited L-wave is increased by *π*, the combined wave from the L-wave and T-wave transmitters is translated upwardly as demonstrated in Fig. 7b. Although the wave that exited the metamaterial consists of both longitudinal and transverse waves, the time-harmonic analysis alone may not clearly distinguish them. However, they are clearly distinguished from the experimental results as shown below.

**Figure 8:**
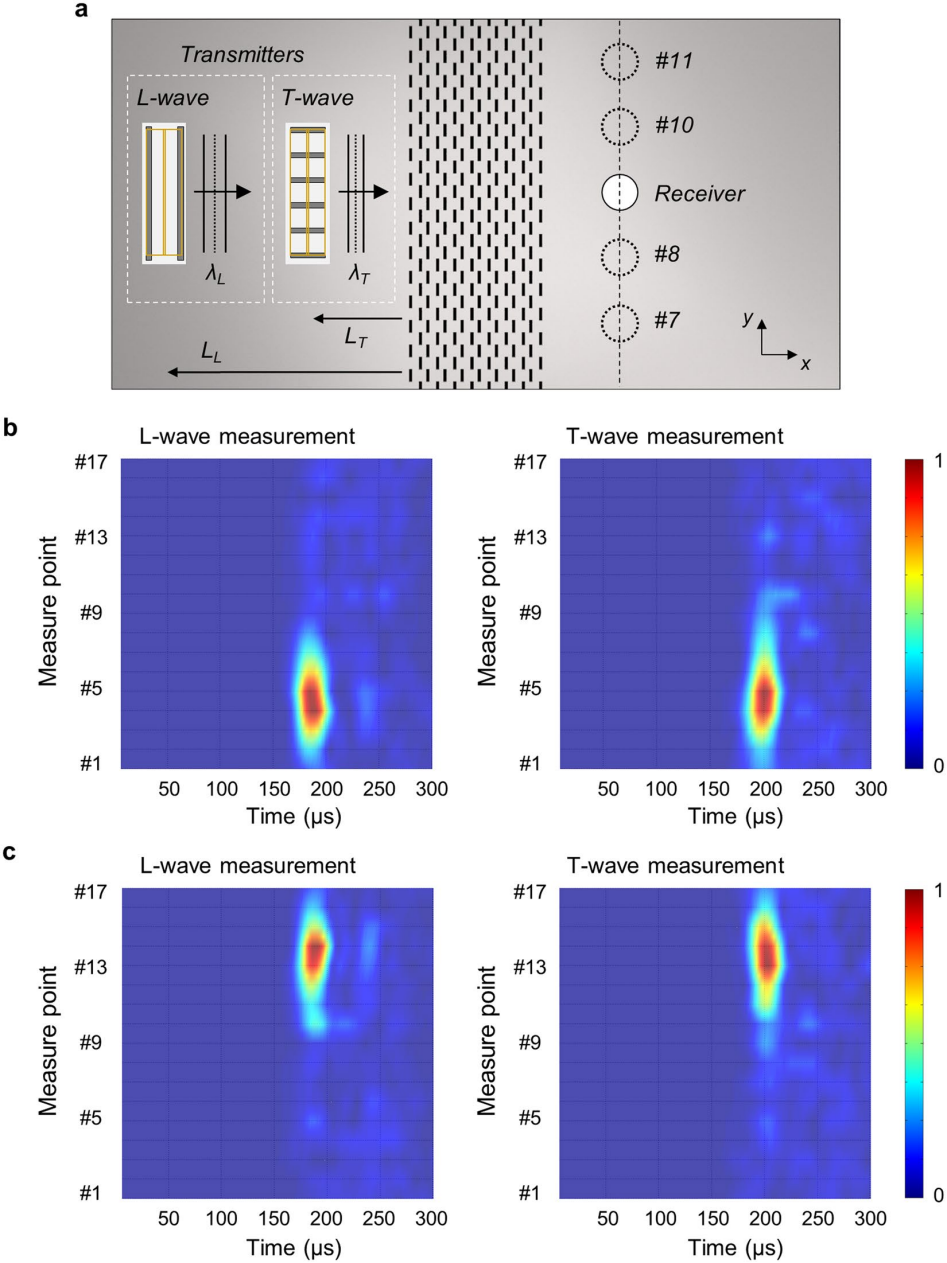
Experimental results of elastic stress wave translations using two types of transmitters, i.e., the L-wave and T-wave transmitters. (**a**) Sketch of experimental measurement system. (**b**) Experimental STFT results for simultaneous excitation of transmitters at 90 kHz longitudinal and transverse waves. Receivers for longitudinal wave (left) or transverse wave (right) are used at every measurement point over time. The signal intensity is the maximum near point #5. (**c**) Experimental STFT results for simultaneous excitation at 90 kHz longitudinal and transverse waves by the two transmitters (See context for the phase control of the transducers). The signal intensity is the maximum near point #13.

The experimental setup is illustrated in Fig. 8a. In essence, the experimental setting is the same as that used to verify the conical refraction phenomenon, except that two transmitters generating the L-wave and T-wave are simultaneously excited with properly-adjusted phase delay between the waves generated by the two transducers. The short-time Fourier transforms of the measured signals at from point #1 to point #17 are plotted in Fig. 8b and c, respectively. The results for the parallel downward and upward translations of elastic waves are presented in Fig. 8b and c, respectively. We use the same phase delays between the two transducers as explained in the numerical simulations. Clearly, the waves mainly pass through the measurement points #5 and #13 for the downward and upward parallel translations, respectively. The results in Fig. 8b and c show that the L-waves arrive earlier than the T-waves because the L-wave speed is faster than the T-wave speed in the base aluminum plate.

## Conclusion

The first realization of conical refraction of elastic waves with a unique anisotropic metamaterial was presented. Due to the existence of multi wave modes in the elastic regime that propagate with different vibration patterns at different wave speeds, the designed metamaterial must form acoustic axes along which the phase velocities of the longitudinal and transverse waves are equal. Since the metamaterial was fabricated in a single-phase isotropic aluminum plate where the longitudinal wave speed is always faster than the transverse wave speed in any direction, the designed unit cell must effectively retard the longitudinal speed more than the shear wave speed. The conical refraction analysis with the designed metamaterial showed that even if the incident wave is a single mode, it is always decomposed into two dissimilar wave modes, longitudinal and transverse, when it exits the metamaterial. Furthermore, the upward and downward exiting wave phases are different depending on the incident mode type, longitudinal or transverse. Thus, it was possible to have either upward or downward parallel translation of elastic waves. To only translate the incident wave up or down, two phase-tuned transducers generating the longitudinal and transverse waves must be used. This strategy may be unique for elastic waves due to the existence of dissimilar wave modes propagating at different wave speeds in isotropic media, which constitute typical specimen. The parallel translation in this investigation can be critically useful when ultrasonic guided wave inspection must be used in structures surrounded by many obstacles. This is not rare in piping and related systems in nuclear power plants. If the working frequency is increased and the metamaterial slab can be further scaled down, the parallel translation involving elastic waves can open a new realm in ultrasonic guided wave inspection.

## Methods

By solving the Christoffel equation^28^ (Γ_*im*_ *k* ^2^ − *ρω* ^2^ *δ*_*im*_)u_*m*_ = 0 with the conditions, *C*_11_ = *C*_66_ and *C*_16_ = *C*_26_ = 0, we can identify the degenerated point of double wavenumbers, equivalently the conical point along the *k*_*x*_ axis. The obtained results are identical to those predicted from the EFC calculated with the finite element method shown in Fig. 2b.

The effective properties of the metamaterial can be determined by approximating the thin three-dimensional plate as a two-dimensional plate under the plane stress. Accordingly, the effective properties in this study are obtained based on the metamaterial unit cell under the plane stress condition.

The aluminum plate used in the experiment has the dimension of 2400 × 1200 × 1 mm^3^. The base plate is large enough to absorb boundary reflection effects. Because a source of 250 mm in length is used to satisfy the plane wave condition, the proposed metamaterial is patterned with a large number (80 × 160) of unit cells; the metamaterial is sufficiently large to accurately check the occurrence of conical refraction and parallel translation.

### Data availability

No datasets were generated or analysed during the current study.

## Electronic supplementary material

Supplementary Information
