# Supplementary material for: Conical Refraction of Elastic Waves by Anisotropic Metamaterials and Application for Parallel Translation of Elastic Waves
Source: Sci Rep. 2017 Aug 30;7:10072. doi: 10.1038/s41598-017-10691-6 (PMC5577280; doi:10.1038/s41598-017-10691-6)
Supplement: Supplementary file 1 — Supplementary Information [file 41598_2017_10691_MOESM1_ESM.doc]

**Supplementary Materials**

**Conical Refraction of Elastic Waves by Anisotropic Metamaterials and Application for Parallel Translation of Elastic Waves**

**Young Kwan Ahn^1^, Hyung Jin Lee^2^ and Yoon Young Kim^1^,[[1]](#footnote-2)**

^1^*School of Mechanical and Aerospace Engineering, Seoul National University,*

*599 Gwanak-ro, Gwanak-gu, Seoul 151-744, Korea*

^2^*Institute of Advanced Machinery and Design, Seoul National University,*

*599 Gwanak-ro, Gwanak-gu, Seoul 151-744, Korea*

**Time transient analysis of the engineered elastic metamaterial**

The results by harmonic analysis were given in the main text. It will be useful to check the results by transient analysis. For the transient analysis, we also used COMSOL Multiphysics. The simulation model used for the time transient analysis is shown in Fig. S1a. The width of the metamaterial slab is 280 mm, and a longitudinal waves (L-wave) was excited some distance ($L_{L}=4\lambda_{L}=238\text{ mm}$) from the wave-incident side of the metamaterial. For an L-wave Gabor pulse centered at 90 kHz, the stress field distribution at $t=30\text{ }\mu s$ is shown in the right side of Fig. S1a. The white dashed box represents the effective medium of the metamaterial. The calculated effective properties at 90 kHz retrieved by the S-parameter retrieval method were used for the simulation. Figure S1a shows that the excited L-waves are incident on the metamaterial at about $t=30\text{ }\mu s$. The time of the wave excitation is set to $t=0\text{ }\mu s$.

Using the simulation results in Fig. S1, the process of wave propagation can be clearly identified both inside and outside the metamaterial slab. In the figure, $(P_{n}\text{)}_{L-wave}$ denotes the power density of an L-wave propagating in the *x* direction. It can be expressed as$(P_{n}\text{)}_{L-wave}=\left|-0.5\text{V}_{x}\sigma_{xx}\right|$ where $\text{V}_{x}$ denotes the particle velocity along the *x* direction and $\sigma_{xx}$, the normal stress. The symbol $(P_{n}\text{)}_{T-wave}$ denotes the power density of a T-wave propagating in the *x* direction with $(P_{n}\text{)}_{T-wave}=\left|-0.5\text{V}_{y}\sigma_{xy}\right|$. Here, $\text{V}_{y}$ represents the particle velocity along the $y$ direction and $\sigma_{xy}$, the shear stress. Figure S1b shows how the normally incident L-wave onto the metamaterial propagates as two longitudinal wave packets (due to the conical refraction) at different time steps, $t=64.5\text{ }\mu s$, $t=124.5\text{ }\mu s$, $t=174.5\text{ }\mu s$ and $t=214.5\text{ }\mu s$. As the incident longitudinal wave reaches the metamaterial slab, two longitudinal wave packets start to deflect into two directions due to conical refraction. Then, the deflected wave packets exit the metamaterial slab in the directions normal to the metamaterial slab surface.

At this point, it should be noted that the doubly-deflected waves inside the metamaterial are coupled longitudinal-transverse waves. Therefore, transverse wave packets also appear and start to deflect into two directions; see Fig. S1c. Because they propagate at the same speed as the speed of the longitudinal wave packets, they all travel exactly at the same speed inside the metamaterial. Accordingly, the regions of high power densities for the longitudinal and transverse wave packets appear exactly at the same time, as can be confirmed in Figs. S1b and S1c. However, the transverse wave packets are retarded as soon as they exit the metamaterial because the transverse wave speed is slower than the longitudinal wave speed in the base isotropic aluminum plate.

The time transient analysis for the case of a normally incident 90 kHz-centered transverse wave (T-wave) pulse onto the metamaterial slab is shown in Fig. S2. It was excited some distance ($L_{T}=4\lambda_{T}=138\text{ mm}$) from the wave-incident side of the metamaterial. Similar observations made for the case of longitudinal wave incidence can be found for the present case. So, the detailed interpretation of the analysis results may be omitted.

It is also useful to know how much power can be transmitted through the metamaterial. By calculating the power transmission through Line 1 and Line 2, one can find the following numerical results:

For incident L-wave 90 kHz Gabor pulse:

$(P_{transmitted})_{L-wave}/(P_{input})_{L-wave}=0.2735$ (S.1a)

$(P_{transmitted})_{T-wave}/(P_{input})_{L-wave}=0.3440$. (S.1b)

For incident T-wave 90 kHz Gabor pulse:

$(P_{transmitted})_{L-wave}/(P_{input})_{T-wave}=0.3249$ (S.2a)

$(P_{transmitted})_{T-wave}/(P_{input})_{T-wave}=0.4301$. (S.2b)

Because the impedance difference between the base aluminum plate and the metamaterial slab, the amount of the power transmission from the base plate to the metamaterial plate is not very high. For practical applications, the impedance mismatch needs to be further improved. This will be a subject of the future study.


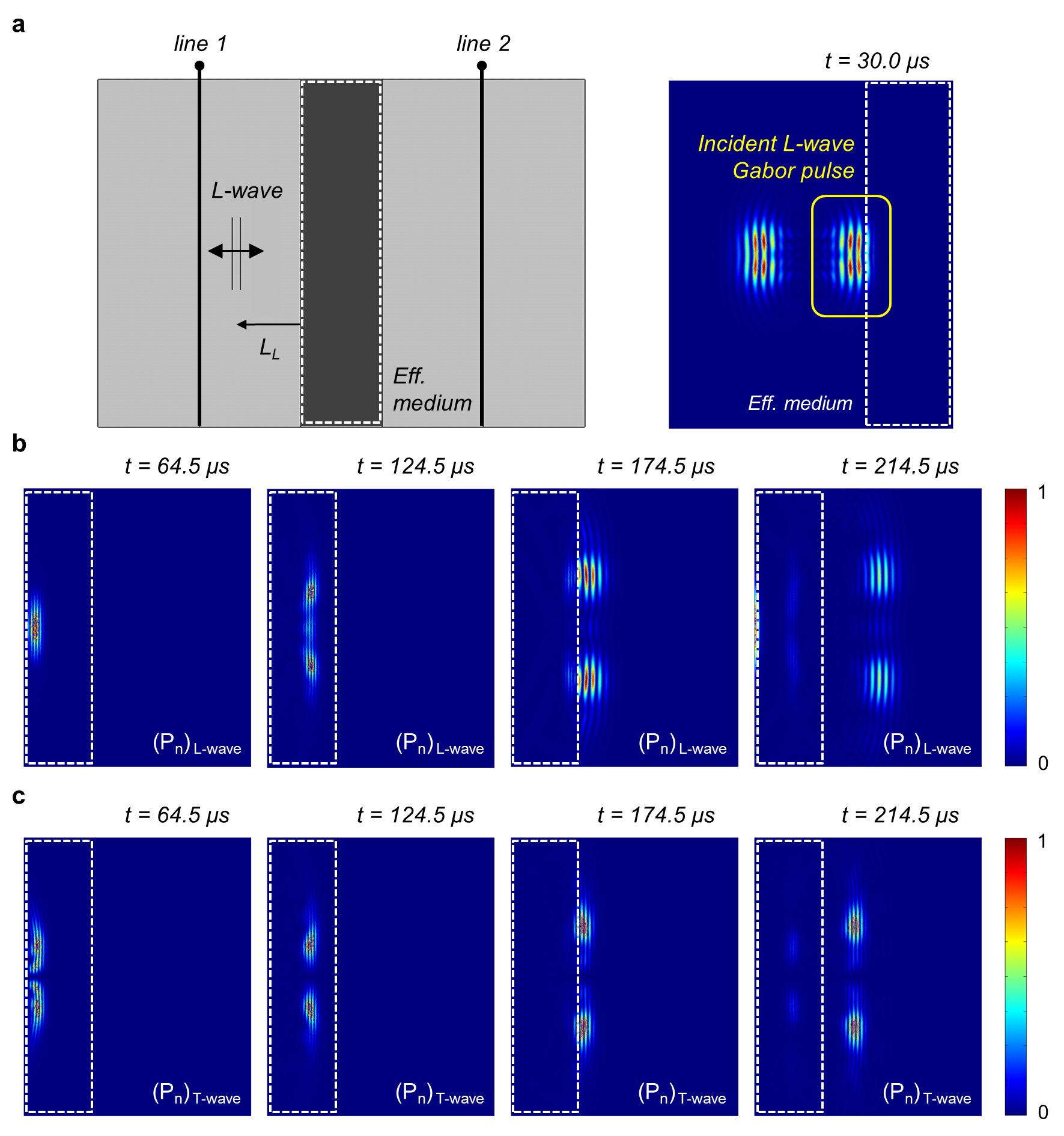


**Figure S1| Time transient analysis of conical refraction with the designed metamaterial slab embedded in an isotropic aluminum plate for an L-wave incidence. (a)** The sketch of the simulation model and the power density distribution at $t=30\text{ }\mu s$ at which the excited L-wave Gabor pulse starts to enter the metamaterial slab. (Another location of high power density denotes waves propagating to the left because the used source is supposed to excite waves both to the right and to the left. Because this wave part is not of any concern, we can ignore it for further analysis.) (**b**) The distribution of the normalized power density of the longitudinal wave component $(P_{n})_{L-wave}$ at different time steps. (**c**) The distribution of the normalized power density of the transverse wave component $(P_{n})_{T-wave}$ at different time steps.


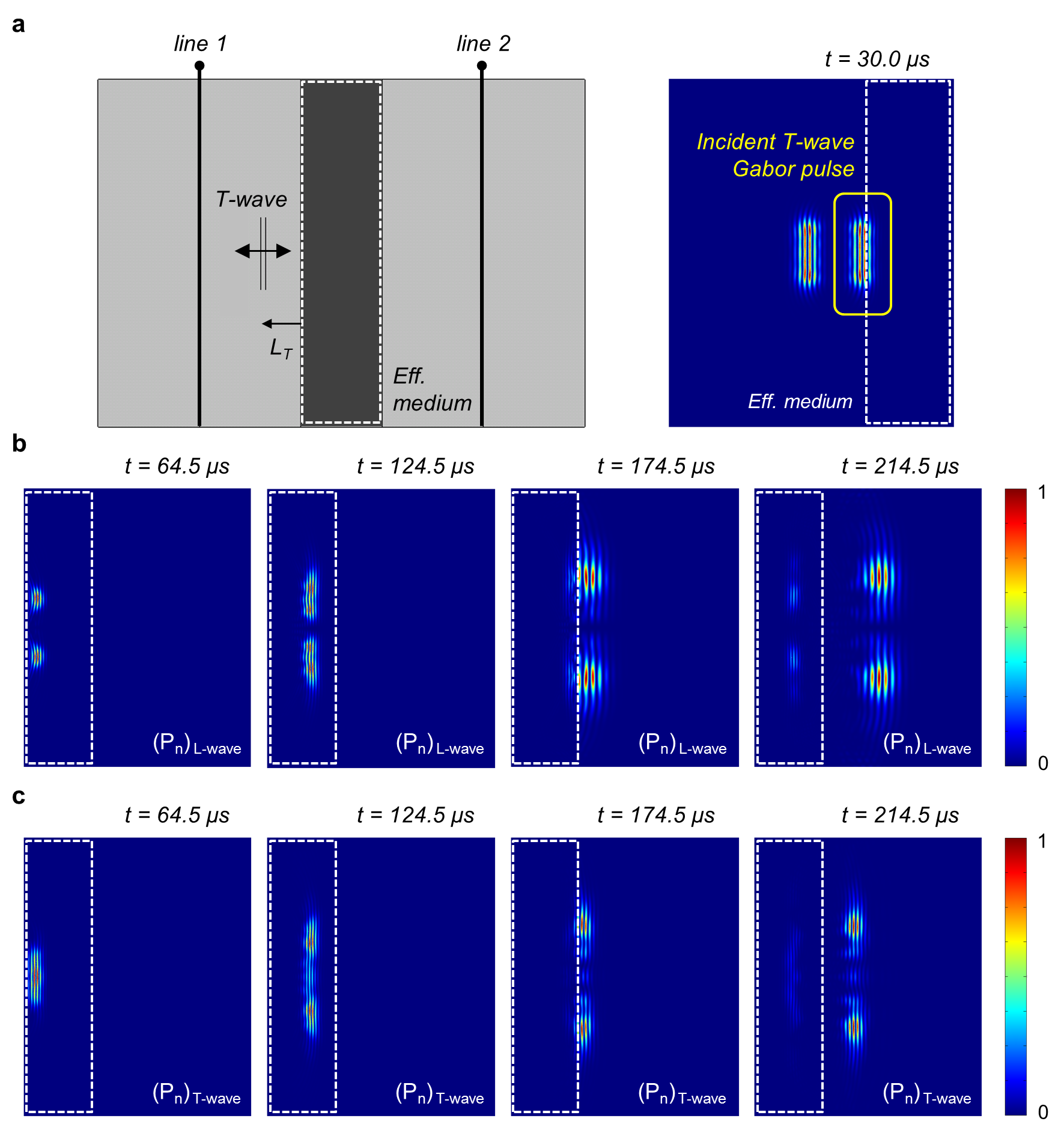


**Figure S2| Time transient analysis of conical refraction with the designed metamaterial slab embedded in an isotropic aluminum plate for a T-wave incidence. (a)** The sketch of the simulation model and the power density distribution at $t=30\text{ }\mu s$ at which the excited T-wave Gabor pulse starts to enter the metamaterial slab. (Another location of high power density denotes waves propagating to the left because the used source is supposed to excite waves both to the right and to the left. Because this wave part is not of any concern, we can ignore it for further analysis.) (**b**) The distribution of the normalized power density of the longitudinal wave component $(P_{n})_{L-wave}$ at different time steps. (**c**) The distribution of the normalized power density of the transverse wave component $(P_{n})_{T-wave}$ at different time steps.

**Deflection angle adjustment**

As explained with Fig. 2b, the deflection angle $\theta_{d}$ can be found from the analysis of the EFC of the metamaterial. The designed metamaterial with the geometric data given in the main text was found to have the deflection angle of $\theta_{d}=37.9^{\circ}$. To change the deflection angle, the curvature of the quasi-longitudinal wave and quasi-transverse wave modes along the $k_{x}$ axis in the EFC should be changed. As explained in the main text, they vary with the anisotropy factor $A=2C_{66}/(C_{11}-C_{12})$, where $C_{ij}$ implies the effective material stiffness components.

Here, we will demonstrate two different deflection angles with different values of the anisotropic factor *A*. We will use the same excitation frequency, 90 kHz, as used in the main text. Compared with the effective material properties ($C_{12}=5.994\times10^{9}$ Pa) used in the main text that yield $\theta_{d}=37.9^{\circ}$, we change only $C_{12}$ with $C_{11}$ and $C_{66}$ unaltered. If we choose $C_{12}=1.994\times10^{9}$ Pa and $C_{12}=9.994\times10^{9}$ Pa, then we obtain $\theta_{d}=30.6^{\circ}$ and $\theta_{d}=44.0^{\circ}$. The simulation results are shown in Fig. S3. Although the specific metamaterial unit cells corresponding to the selected effective properties are not designed here, the numerical results show that the deflection angles can be adjusted if proper effective properties are chosen.


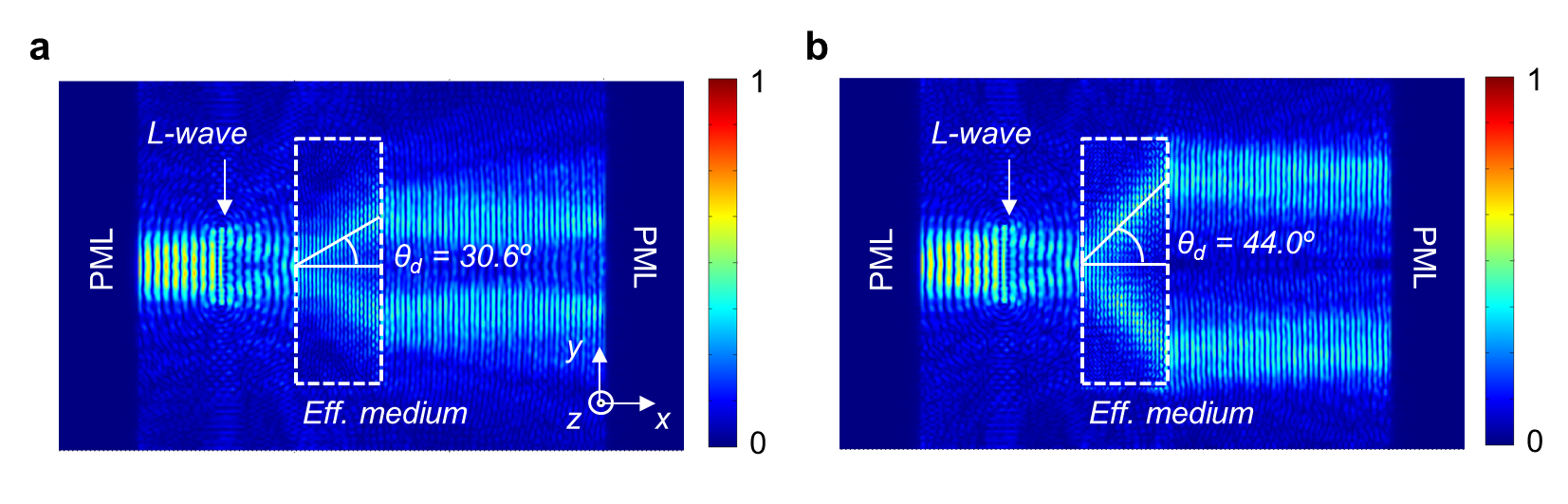


**Figure S3| Deflection angle (**$\theta_{d}$**) adjustment by changing effective material properties.** Normalized stress field distributions and the deflection angles are shown for media with varied $C_{12}$ with other $C_{ij}$’s given in Table 1 for 90 kHz: (**a**) $C_{12}=1.994\times10^{9}$ Pa and (**b**) $C_{12}=9.994\times10^{9}$ Pa. The simulation results are obtained for the incidence of a longitudinal wave of the 90 kHz Gabor pulse type. The white dashed boxes cover the regions of anisotropic media with the suggested material properties.

**Experiment procedure: wave excitation and measurement**

Fig. S4a shows an overview experimental setup. Fig. S4b shows the equipment used to perform the experiments for this study. A function generator (Agilent Technologies 33220A) was used to excite wave sources of different pulses, such as the Gabor, sine, and rectangular pulses. The generated signals were then amplified by a power amplifier (T&C Power Conversion AG1017L) before entering the transmitter made of the Magnetostrictive Patch Transducer (MPT). The MPT transmitter generates an elastic strain in the plate, which propagates through the plate to which the transmitter is attached to. The receiver, made of another type of MPT, picks up the strain as a voltage output signal, which is then amplified by a pre-amplifier (Stanford Research Systems SR560). The amplified signal is sent to an oscilloscope.

Two sets of MPT receivers were used: one set to measure the longitudinal wave, i.e., the S_0_ wave propagating in the plate and the other set to measure the transverse wave, i.e., the SH_0_ wave propagating in the plate. The measured raw data are shown in Fig. S5 where the incident wave signal is a 90 kHz longitudinal wave of the Gabor pulse type. These data are used to obtain the Short-time Fourier transform (STFT) results shown in the main text.

*
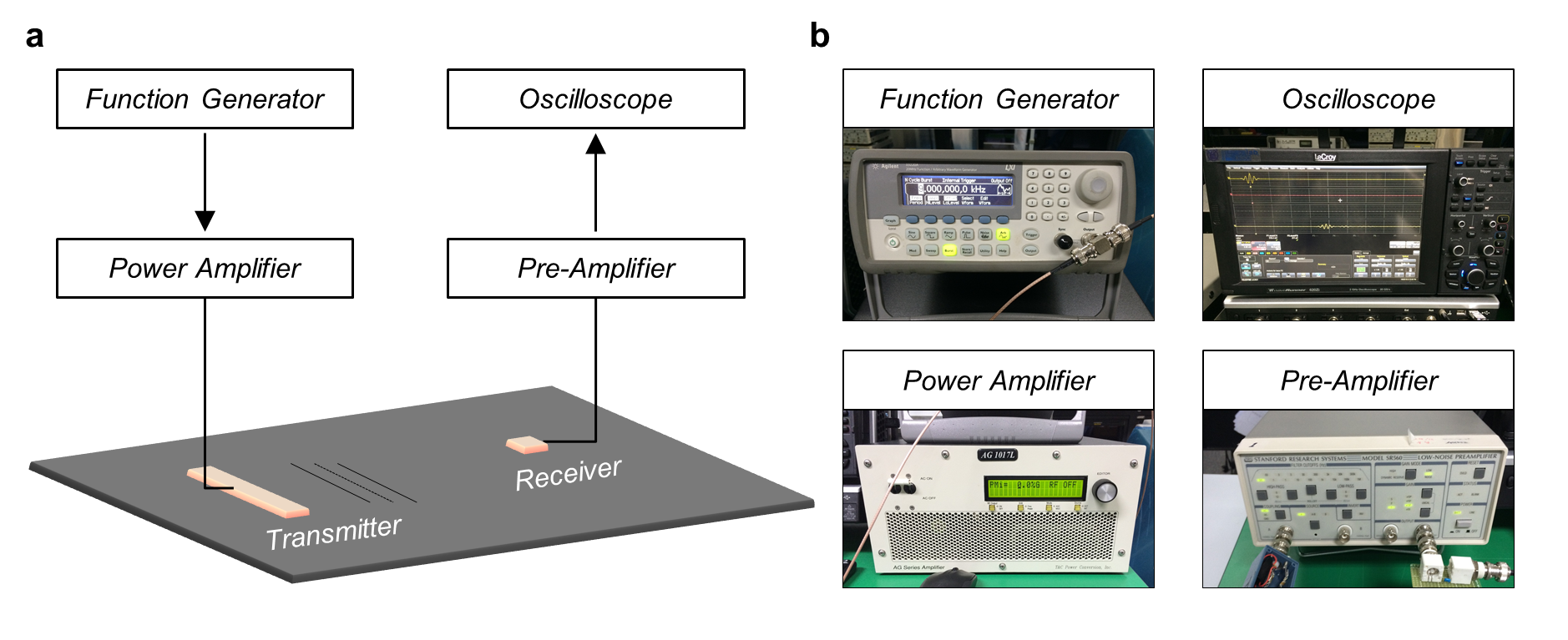
*

**Figure S4| Experimental setup for wave generation and measurement. (a)** Schematic diagram of the experiment. **(b)** Used experiment equipment: function generator, power amplifier, pre-amplifier, oscilloscope.

*
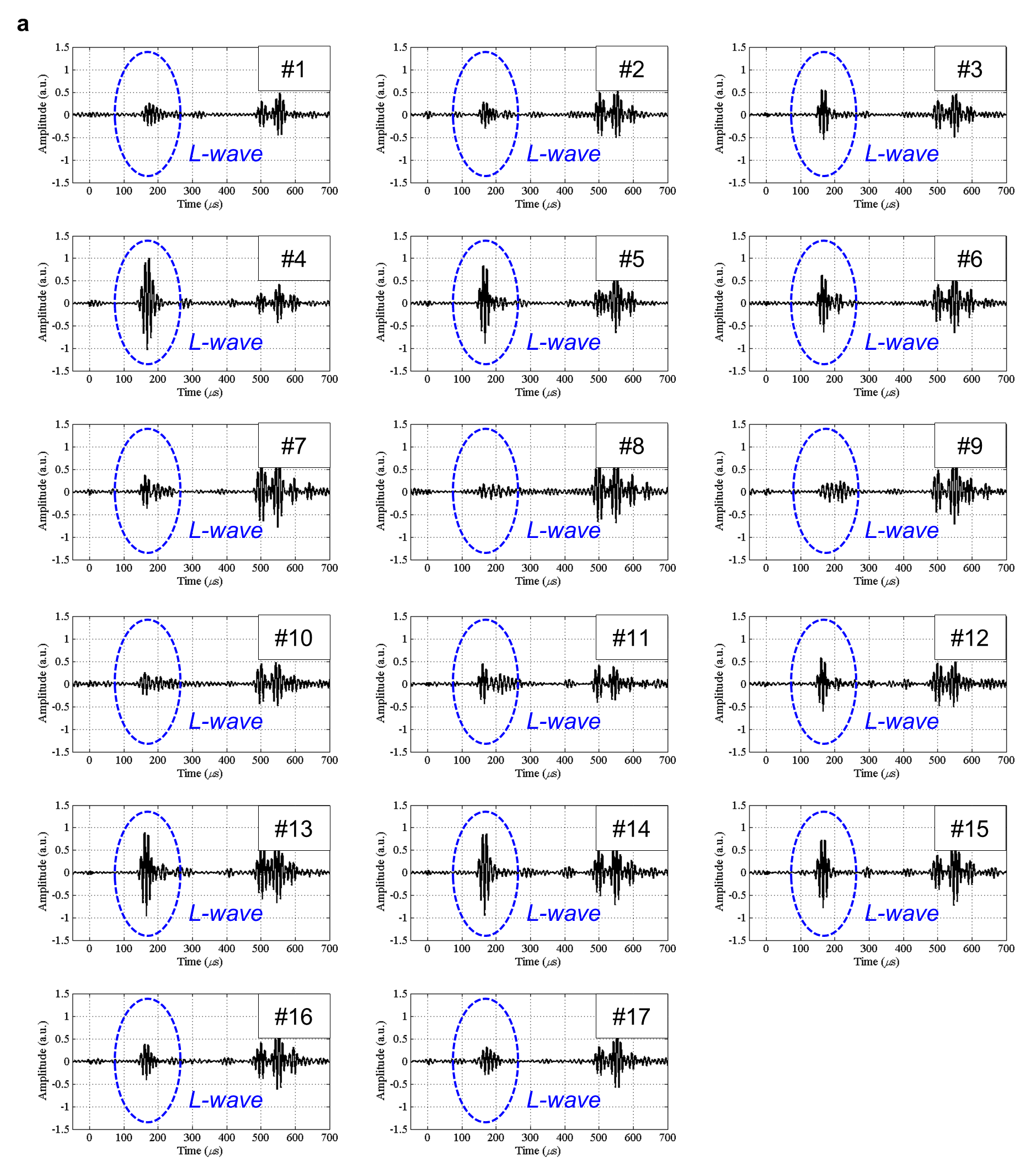
*

**
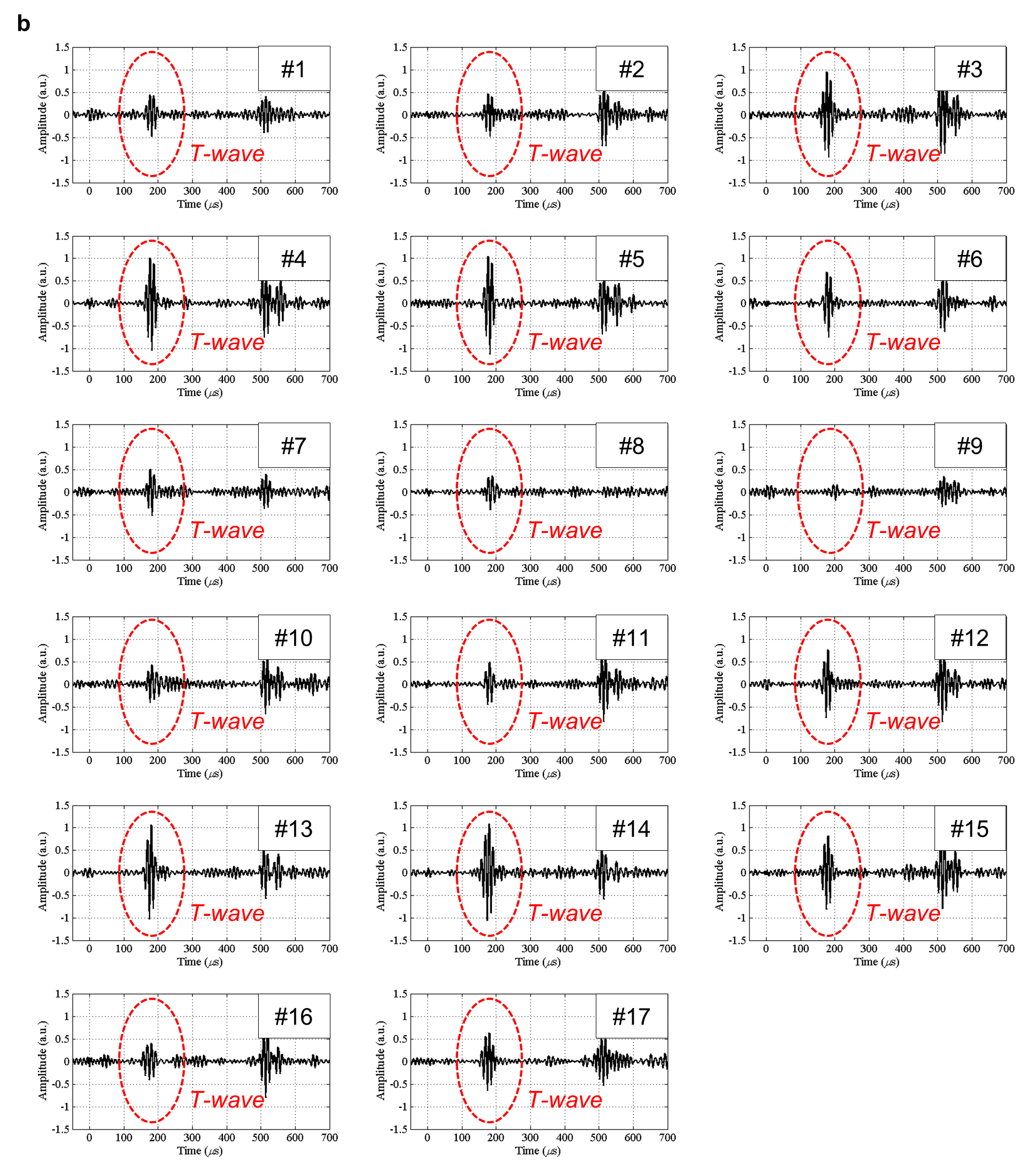
**

**Figure S5| The transient signals measured at various locations along the line A-B marked in Fig. 3 for an incident longitudinal (S_0_) Gabor wave pulse. (a)** Measured wave mode: longitudinal (S_0_) and (**b**) measured wave mode: transverse (SH_0_).

**Performance of conical refraction over a wide frequency range**

Because the proposed metamaterial is a non-resonant type, it is supposed to operate over a wide range of frequency. The actual metamaterial design was performed for 90 kHz, but the metamaterial realizes the conical refraction satisfactorily in a wide range, say, from 60 kHz to 120 kHz. Figures S6 and S7 shows the experimental results demonstrating how well the designed metamaterial realizes the phenomenon of conical refraction at 60 kHz and 120 kHz, respectively. The measurement locations used to prepare Figs. S6 and S7 are identical to those used for the results obtained in the main text at 90 kHz. The results in Fig. S6 and S7 clearly demonstrate that the conical refraction phenomenon appears distinctly both at 60 kHz and 120 kHz, confirming the wide-frequency operating capability of the designed metamaterial.


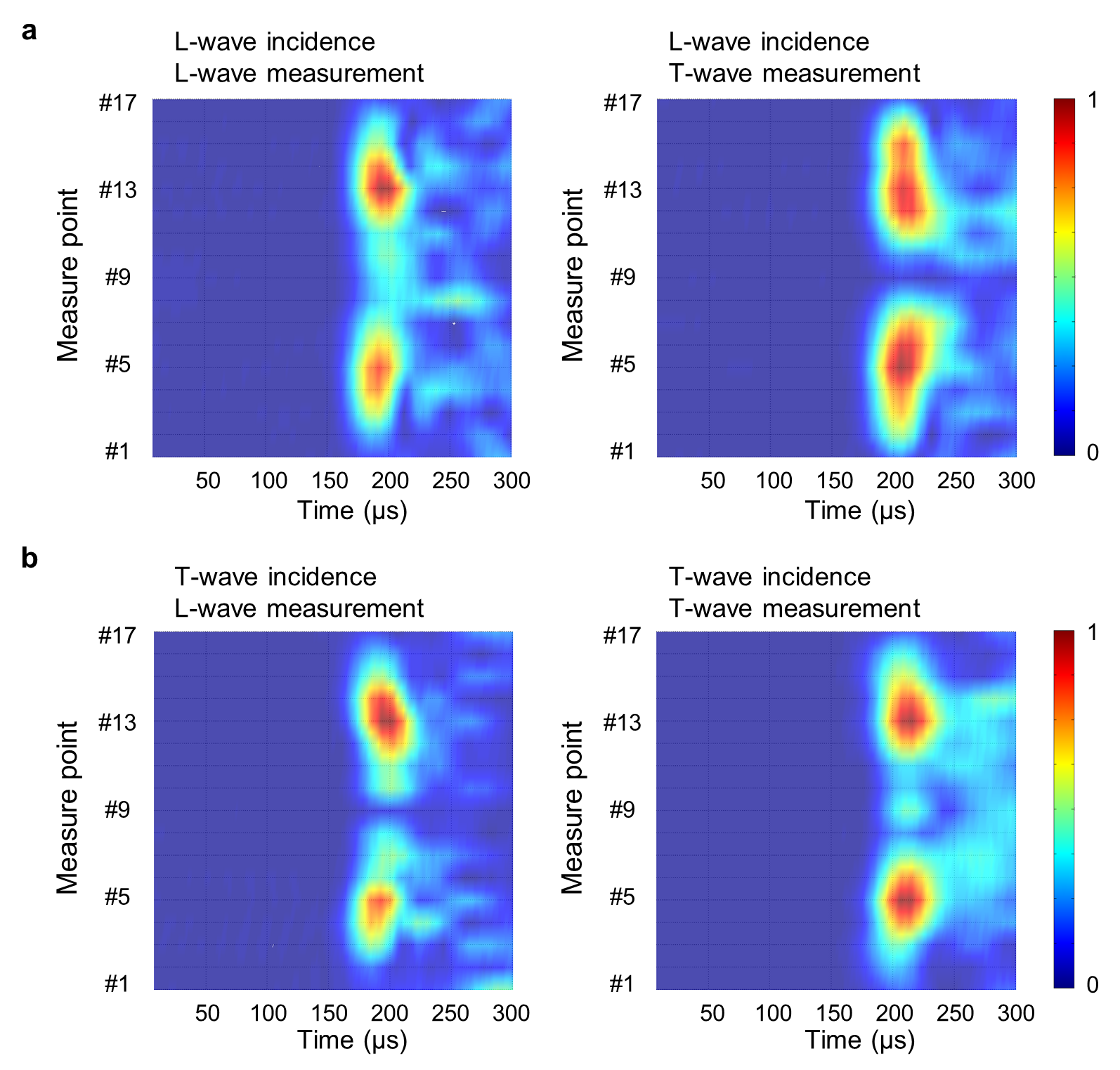


**Figure S6| Short-time Fourier transforms (STFT’s) of the measured signals by the receivers. (a)** A 60 kHz Gabor pulse longitudinal wave incidence. Left: STFT of the measured longitudinal wave, Right: STFT of the measured transverse wave. **(b)** A 60 kHz Gabor pulse transverse wave incidence. Left: STFT of the measured longitudinal wave, Right: STFT of the measured transverse wave.

**
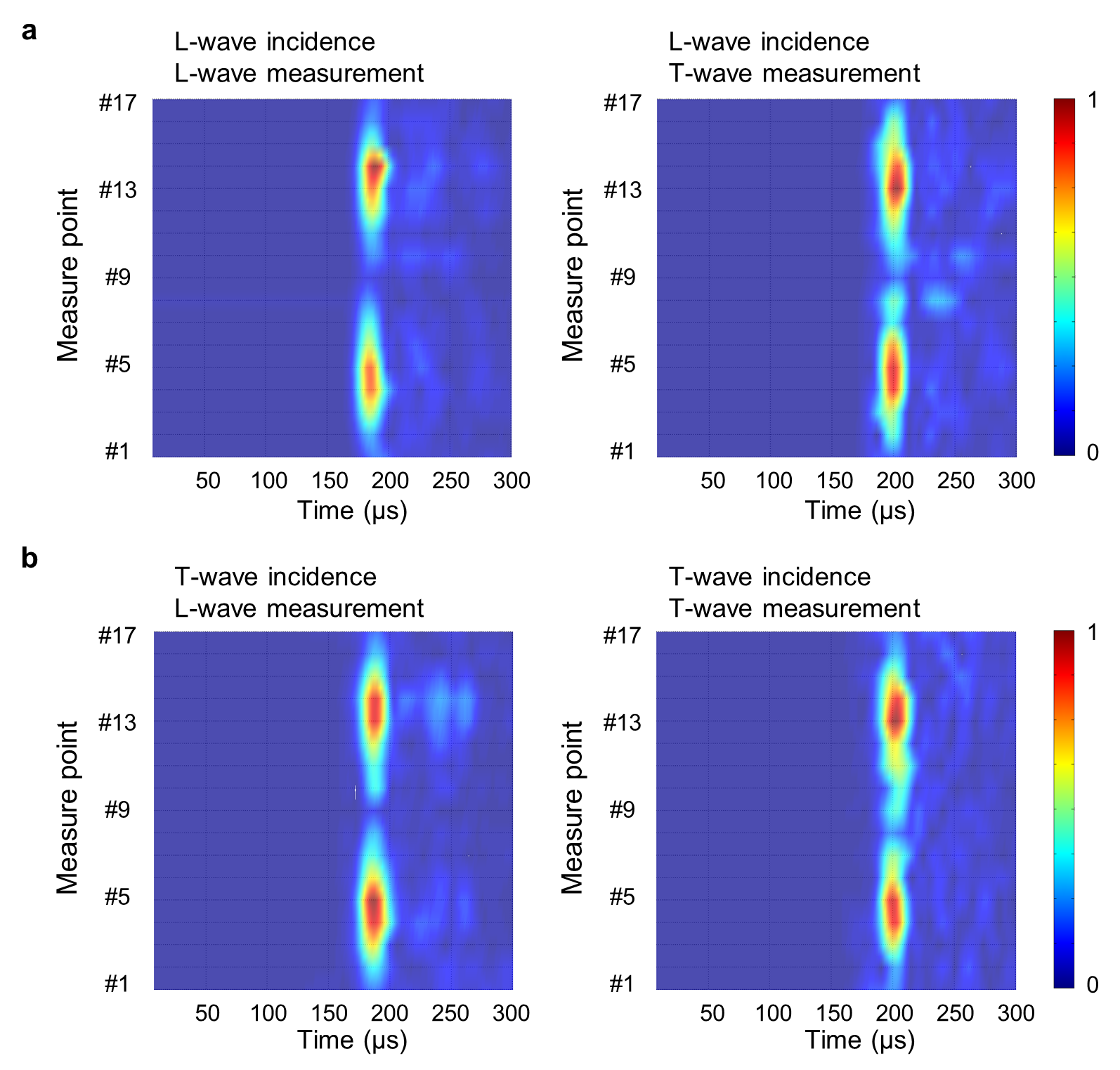
**

**Figure S7| Short-time Fourier transforms (STFT’s) of the measured signals by the receivers. (a)** A 120 kHz Gabor pulse longitudinal wave incidence. Left: STFT of the measured longitudinal wave, Right: STFT of the measured transverse wave. **(b)** A 120 kHz Gabor pulse transverse wave incidence. Left: STFT of the measured longitudinal wave, Right: STFT of the measured transverse wave.

**Demonstration of wave detouring or cloaking**

An interesting application of the phenomenon of conical refraction would be to hide an object by detouring the path of propagating waves. This wave detouring or cloaking can be realized if two flat metamaterial slabs are placed before and after an object to hide or avoid.

As an object to hide or an obstacle along the wave path, we choose a rectangular hole sized of 100$\times$200 mm^2^ in a plate, as illustrated in Fig. S8a. As before, we will use a plate made of aluminum. When an elastic wave is incident onto the hole, it will be reflected by the hole. As an illustration, we will simply use the incidence of a 90 kHz transverse wave for the present simulation, but all the arguments made with the T-wave incidence case will be valid for the incidence case of a longitudinal wave. If two flat metamaterial slabs are installed before and after the hole, the incident wave onto the hole detour around it, as illustrated in the simulation results in Fig. S8b. It clearly shows that the incident wave can detour the rectangular void because the used metamaterials exhibit conical refraction. For the simulation, the distance between the wave source and the wave-entering side of the left metamaterial slab is 100 mm. The size $L_{MM}$ of the metamaterial and the gap $L_{C}$ between the two metamaterial slabs are given in the caption of the figure. (Because there is impedance difference between the base material and the metamaterial, the full wave transmission from the wave incident side to the wave exiting side cannot be fulfilled. This issue was also remarked in the main text.) Also, the present wave detouring works only for unidirectional waves propagating along a specific direction (the horizontal direction in the present case). This problem should be also addressed if omnidirectional wave incidence needs to be considered. Nevertheless, this example demonstrates a possibility of wave detouring or cloaking by using the phenomenon of conical refraction.


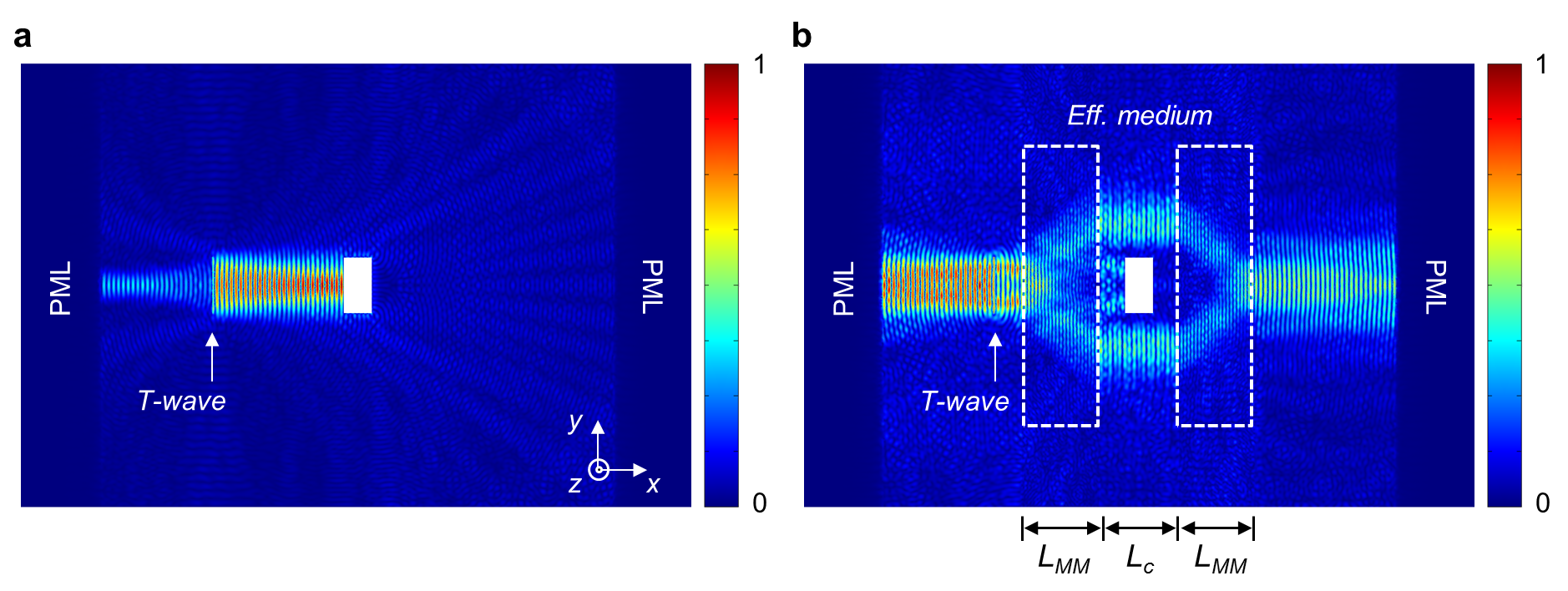


**Figure S8| Numerical simulations of elastic wave detouring around a rectangular hole sized of $100\text{ }\times200$ mm^2^ or cloaking by a set of elastic anisotropic metamaterials demonstrating conical refraction. (The incident wave is a 90 kHz transverse wave.) (a)** Normalized stress distribution when there is no metamaterial installed and **(b)** normalized stress distribution when two metamaterial slabs are installed before and after the rectangular hole ($L_{MM}=286.2\text{ mm}$ and $L_{C}=280\text{ mm}$). White dashed boxes surround the anisotropic elastic metamaterial slabs with the calculated effective material properties in Table 1.

1. Corresponding Author, Professor, [yykim@snu.ac.kr](mailto:yykim@snu.ac.kr), phone +82-2-880-7154, fax +82-2-872-1513 [↑](#footnote-ref-2)
